# Gut Microbiome, Diet and Depression: Literature Review of Microbiological, Nutritional and Neuroscientific Aspects

**DOI:** 10.1007/s13668-025-00619-2

**Published:** 2025-02-10

**Authors:** Laura Clerici, Davide Bottari, Benedetta Bottari

**Affiliations:** 1 Department of Food and Drug, University of Parma, Parma, Italy; 2 IMT School for Advanced Studies, Lucca, Italy; 3 Department of Food and Drug, University of Parma, Parma, Italy

**Keywords:** Gut microbiota, Diet, Gut-brain axis, Depression

## Abstract

**Purpose of Review:**

This review explores the intricate relationships among the gut microbiota, dietary patterns, and mental health, focusing specifically on depression. It synthesizes insights from microbiological, nutritional, and neuroscientific perspectives to understand how the gut-brain axis influences mood and cognitive function.

**Recent Findings:**

Recent studies underscore the central role of gut microbiota in modulating neurological and psychological health via the gut-brain axis. Key findings highlight the importance of dietary components, including probiotics, prebiotics, and psychobiotics, in restoring microbial balance and enhancing mood regulation. Different dietary patterns exhibit a profound impact on gut microbiota composition, suggesting their potential as complementary strategies for mental health support. Furthermore, mechanisms like tryptophan metabolism, the HPA axis, and microbial metabolites such as SCFAs are implicated in linking diet and microbiota to depression. Clinical trials show promising effects of probiotics in alleviating depressive symptoms.

**Summary:**

This review illuminates the potential of diet-based interventions targeting the gut microbiota to mitigate depression and improve mental health. While the interplay between microbial diversity, diet, and brain function offers promising therapeutic avenues, further clinical research is needed to validate these findings and establish robust, individualized treatment strategies.

## Introduction

A link between diet and mental health has been long suggested [1, 2]. This is quite logical, considering that the brain’s composition, structure, and function depend on the availability of appropriate nutrients [3, 4]. The intake and quality of food have an impact on brain function, indicating diet as a powerful tool for improving mental health, mood, and cognitive performance [5, 6]. A further puzzle piece is the link between what we eat and the gut microbiota, as understanding the mechanisms beyond that complex interplay would add knowledge on its impact on mental health or disorders such as depression [7]. Recent studies have explored how dietary patterns and microbiota composition influence brain function, mood, and the risk of developing mental health disorders such as depression [8–11]. This article delves into the intricate communication pathways between the gut and brain, emphasizing microbiological, nutritional, and neuroscientific aspects.

### The Human Microbiota

The human microbiota is defined as the set of microorganisms which live in symbiosis with the human body [12] including fungi, protozoa, viruses and bacteria; it is not exclusive of the intestine but spread in several parts of the body [13]. The intestinal microbiota is the most densely populated, consisting of microorganisms interacting with each other through different relationships and the outcome of the interaction is also influenced by the host’s physiology [14]. The definition of microbiota should be completed by the concept of microbiome, defined as the genetic heritage of microbiota, that means the totality of the genes expressed by the microorganism, including all the environment [14]. Microbiota is composed of microorganisms estimated to outnumber human body cells by a factor of 1.5 [15]; the number of different genes present at the level of the ecosystem is responsible for the biodiversity of the microbiome and is related to richness [16]. This is also linked to the resilience of microbiota, i.e. the ability to respond and react to changes, reorganizing itself in such a way as to keep unchanged the functions, composition, and initial structure, guaranteeing functional stability and homeostasis [17].

The functions of the microbiota are numerous and can be directly and indirectly related to the health and well-being of the individual; they can be classified as: i) Protective functions: resistance to colonization by pathogens [18], activation of innate and adaptive immunity; regulation of inflammatory cytokines; promotion of immune system development [19]; barrier through the production of antimicrobial proteins including bacteriocins mostly lead by lactic bacteria that are able to eliminate entero-invasive pathogens that could alter eubiosis [20]; ii) Metabolic functions: fermentation of non-digestible substrates; production of short chain fatty acids (SCFA), which modulate intestinal inflammation and protect mucosa integrity [21, 22]; influence on energy metabolism and body weight; production of B and K vitamins [23]; biosynthesis of amino acids; biotransformation of bile acids [24]; iii) Structural functions: growth, differentiation, and regulation of intestinal epithelial cells; development of intestinal villi and crypts; support of integrity and modulation of intestinal barrier permeability [25].

The gut microbiota is composed in adults mainly by anaerobic bacteria from the major phyla of Firmicutes (predominantly *Lachnospiraceae* and *Ruminococcaceae*), Bacteroidetes, Actinobacteria, Proteobacteria, and Verrucomicrobia (*Akkermansia*) [26]. The main metabolic pathway used by bacteria is fermentation, although some of them can use acetogenesis, and others are endowed with sulphate-reducing enzymes, while methanogenesis is exclusive to Archaea [27]. We can describe some species as saccharolytic or proteolytic depending on their ability to use substrates such as carbohydrates, producing SCFA, or proteins, producing branched chain fatty acid (BCFA), SCFA to a lesser extent, and phenolic compounds [21, 22, 28, 29].

### Overview of the GUT-Brain Axis

**Fig. 1:**
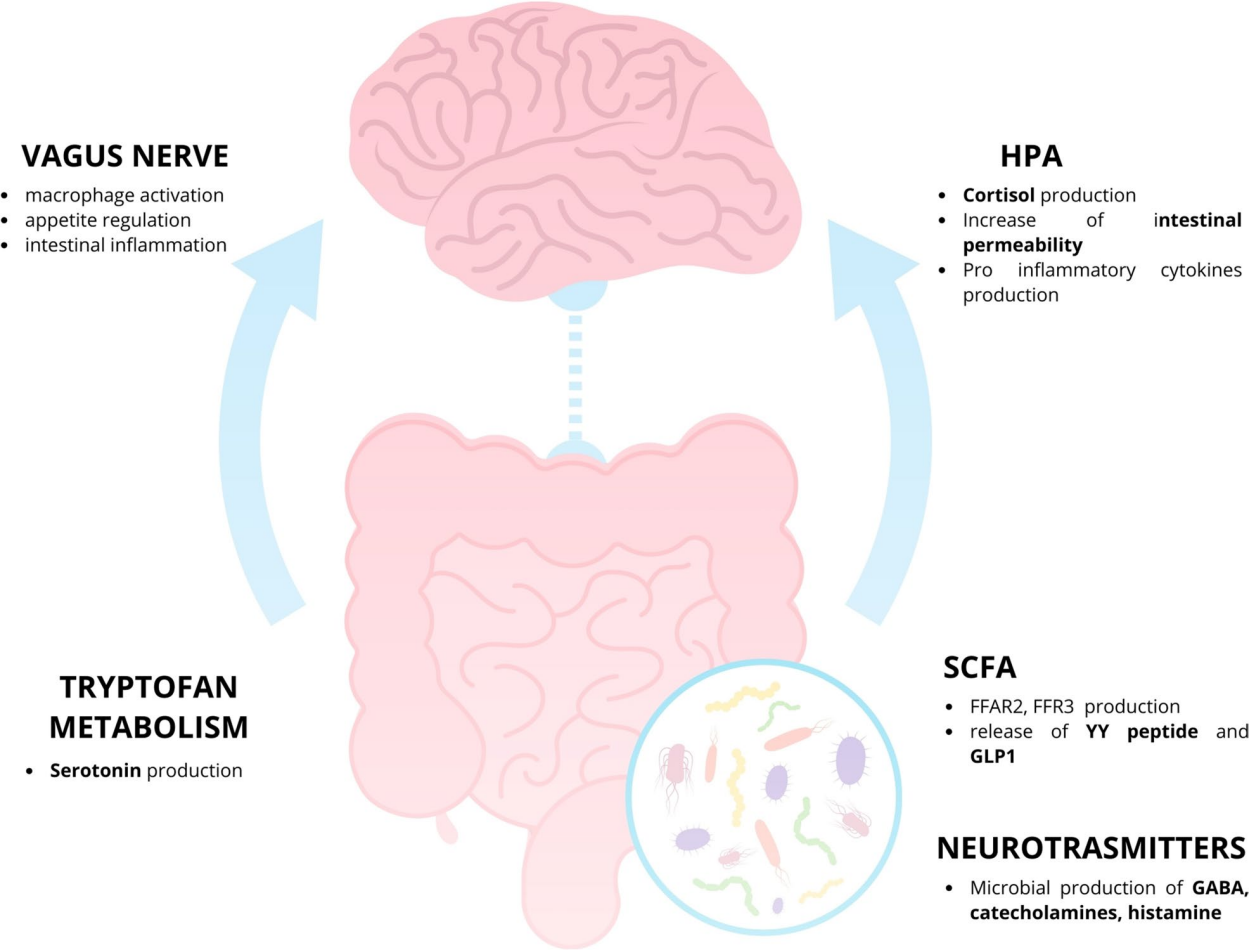
Bidirectional homeostatic communication pathway along intestinal-brain axis uses neural, hormonal, and immunological pathways. Figure created with Canva

The gastrointestinal tract (GIT) and the brain are intricately connected through the gut-brain axis [30], a bidirectional homeostatic communication network involving neural, hormonal, and immunological pathways (Fig. 1). Dysfunction of this axis has been linked to several pathologies, offering insights into potential therapeutic strategies [10]. The gut-brain axis integrates signals from various systems, including the neuroendocrine, autonomic nervous system (ANS), and enteric nervous system (ENS) [30, 31]. The ENS, located in the GIT, comprises approximately 500 million nerve endings and represents the largest concentration of immune cells in the body, facilitating communication with the brain via the vagus nerve [32, 33]. Immune cells in the gut release cytokines, essential for inflammatory responses, while neuroendocrine hormones like cortisol alter intestinal permeability and influence cytokine secretion [10]. This two-way communication enables brain signals to regulate intestinal motor, sensory, and secretory functions, while gut signals influence brain activity [34].

### The Role of the Intestinal Microbiome in Neurological Processes

The intestinal microbiome has emerged as a critical regulator of host physiology [34, 35] and central nervous system (CNS) function [10, 36, 37], highlighting the concept of the microbiome-intestine-brain axis [38]. Disruptions in the delicate balance of the microbiome have been linked to psychopathologies, which is particularly significant given the microbiome's susceptibility to external factors, including diet [39]. The main communication routes of the gut-brain axis include: i) the vagus nerve; ii) microbial metabolites; iii) tryptophan metabolism; iv) the hypothalamic–pituitary–adrenal axis (HPA); and v) neurotransmitters.

## Key Communication Pathways of the Gut-Brain Axis

### Vagus Nerve

The vagus nerve serves as a primary communication channel between the gut and brain, transmitting sensory and motor information [40]. It responds to mechanical, chemical, and hormonal signals through its diverse receptors [41]. The vagus nerve also modulates pro-inflammatory cytokine levels via the vaso-vagal anti-inflammatory reflex [42], a mechanism implicated in conditions such as inflammatory bowel disease [43]. Strengthening these observations, the link between the modulation of vagus nerve activity and communication between the gut and the brain, such as appetite regulation, intestinal inflammation, and mood, has also been repeatedly investigated (see [44] for a review). Interestingly, in recent years, devices that allow the stimulation of the vagal nerve (VNS) [45] via transcutaneous stimulation were developed, allowing for a causal alteration of gut-brain interplay. Transcutaneous VNS (tVNS) electrically modulates the auricular branch of the vagus nerve. It is a simple, non-invasive technique leading to the stimulation of subcortical nuclei like the locus coeruleus and the nucleus of the solitary tract, ultimately resulting in cortical activation. tVNS, has been reported to increase neurotransmitter levels such as norepinephrine and gamma-aminobutyric acid (GABA), favoring improvements in related cognitive performance [46]. tVNS has been recently employed to foster brain plasticity, alter functioning, and reduce diseases such as depression [47, 48]. Elger and colleagues employed VNS in epilepsy patients, monitoring their mood for six months. Results highlighted positive effects on mood after tVNS treatment, which were sustained up to the following 6-month [48].

### Microbial Metabolites

Short-chain fatty acids (SCFAs)—including propionate, butyrate, and acetate—are key microbial metabolites produced in the gut. They are derived from the metabolism of certain microbial genera, including *Eubacterium, Roseburia, Faecalibacterium, Bifidobacterium, Lactobacillus* and *Enterobacter* [49]. SCFAs activate several G-protein coupled receptors (GPCRs), of which free fatty acid receptor 2 (FFAR2, designated GPR43) and 3 (FFAR3, designated GPR41) are the most investigated [50–52]. Expression of both receptors has been reported in the colon, various immune cells, and the heart. Only FFAR2 is expressed in adipocytes and skeletal muscle, while FFAR3 is expressed in the peripheral nervous system and blood–brain barrier (BBB) [53, 54]. No expression of FFAR2 has been reported in the brain [55]. SCFAs exert multiple beneficial effects. Several studies showed that SCFAs stimulate the assembly of tight junctions [56, 57], modulate immune cells [58], regulate chemotaxis, the inflammatory process by neutrophils [59, 60], suppress cytokine production by myeloid cells [61], regulate T helper 1 lymphocyte activity, and T helper 17 cell differentiation [62, 63]. Overall, SCFAs influence the immune response by acting with regulatory action and activating anti-inflammatory pathways. SCFAs are part of the first line of defense between the microbiota and the permeability of the host intestinal barrier by enhancing the mucosal barrier by stimulating mucus production, which is probably mediated by FFAR3 [64]. In addition, SCFAs play a role in enteroendocrine signaling by binding to a related receptor (e.g., GPR43 or GPR41) to stimulate the release of neuropeptides, such as YY peptide (PYY) and glucagon-like peptide (GLP-1), which influence the regulation of energetic homeostasis through activation of both enteric and primary afferent vagal pathways [65]. Finally, they can stimulate the secretion of the neurotransmitter 5-HT (5-hydroxytryptamine) in the intestinal lumen as well as in the vascular system [66], which is an extremely important factor in the regulation of intestinal-brain communication regulating human behaviour; about 90% of serotonin is produced in enterochromaffin cells in the gastrointestinal tract and by some microbiota genera such as *Escherichia *spp and *Enterococcus s*pp [67].

### Tryptophan Metabolism

Tryptophan is an essential amino acid and a precursor of many biologically active agents, including serotonin, which has been traditionally associated in depressive disorders as it is involved in the regulation of mood, sleep/wake rhythm, sexual functions, and appetite [68–70]. Serotonin is predominantly found in the intestine, where it is synthesized by tryptophan in enterochromaffin cells of the GIT [71]. Serotonin synthesis is highly dependent on the availability of tryptophan and the tryptophan hydroxylase enzyme (TPH), that is a rate-limiting enzyme in the biosynthesis of the neurotransmitter. Low plasma tryptophan levels were associated with impaired immune function [72, 73]. The dominant physiological pathway for tryptophan metabolism is the kynurenine pathway, which accounts for over 95% of the peripheral tryptophan available in mammals [74], and its alteration has been implicated in many brain and gastrointestinal disorders. Kynurenine can be further metabolized in two different products, quinolinic acid which produces several neurotoxic metabolites and kynurenic acid which has a neuroprotective role. Indeed, the increased conversion of plasma kynurenine to kynurenic acid has been proposed as neuroprotective and attenuating stress-induced depression [75]; there is some evidence suggesting that probiotics such as *Bifidobacterium infantis* [76] may lead this conversion [76]. Certain mediators of inflammation and corticosteroids may induce the action of certain enzymes, such as indoleamine-2, 3-dioxigenase or tryptophan 2, 3-dioxigenase which limit the rate of the hepatic kynurenine metabolic cascade with neurological consequences [77].

### Hypothalamic–Pituitary–Adrenal Axis

The Hypothalamic–Pituitary–Adrenal Axis (HPA) is one of the main neuroendocrine systems within the human body, better known as the main neuroendocrine coordinator in response to stress [78] and one of the main non-neuronal pathways of communication on the microbiota-intestinal-brain axis. When altered homeostasis occurs, corticotrophin releasing factor (CRF) is produced from the paraventricular nucleus of the hypothalamus (PVN), stimulating the release of adrenocorticotropic hormone (ACTH) from the anterior pituitary gland. This hormone is released into the systemic circulation and targets the adrenal cortex, resulting in the release of glucocorticoids [79], which in the brain interact with high-affinity mineralocorticoid receptors and low affinity glucocorticoid receptors [80, 81]. The main function of the activation of the HPA axis is precisely to prepare the body for the "fight-or-flight" response [82]: one of the main outputs is the negative feedback in which glucocorticoids act on the hypothalamus and pituitary gland inhibiting adrenal secretion. At the same time, PVN activity is regulated by multiple afferent circuits: sympathetic, parasympathetic, and limbic [83]. The HPA axis also interacts with other non-neural pathways connecting the gut and brain, including the vagus nerve: in rodents, vagal stimulation increased CRF mRNA expression in the hypothalamus [83], and plasma levels of ACTH and corticosterone were surprisingly high after vagal stimulation. The interactions of the immune system-HPA axis are implicated in several stress-related and inflammatory disorders: it has been seen in animal models how psychological stress can increase intestinal permeability, inducing bacterial translocation in the host [84]. The activation of the immune response of the mucosa through exposure to bacteria and antigens induces the secretion of pro-inflammatory cytokines, which, in turn, activate the HPA axis, highlighting once again how the microbiota plays a key role in this relationship as well.

### Neurotransmitters

Several bacterial genera (*Lactobacillus* spp, *Bifidobacterium* spp, *Escherichia* spp, *Enterococcus* spp) were shown to produce neurotransmitters and neuropeptides including GABA, serotonin, catecholamines, and histamine [85, 86]. Neurotransmitters are chemical messengers that transmit signals through a chemical synapse from one neuron to another target neuron, muscle cell, or glandular cell. Neuropeptides are small proteins that can be released in the brain to activate different receptors, allowing neurons to communicate with each other [87].

## Gut Microbiota and Depression

Depression is a leading cause of disability in the world, affecting 4.4% of the world's population [88]. Major Depressive Disorder (MDD) is the most prevalent manifestation of depression, including a reduction of Brain-Derived Neutrophic Factor (BDNF), which rules neurons survival [89]; an increase in pro-inflammatory cytokines [90], and elevated levels of stress-related hormones [91]. These hormonal changes activate the HPA axis, leading to its hyperactivation, which is further associated with depression [92]. Effective therapies for depression reduce or cancel the increase in the inflammatory response and limit the activation of the HPA axis [92, 93]. In rodent, stress has been indicated as a factor that can alter the function of the intestinal barrier, allowing lipopolysaccharides and other molecules to enter the bloodstream, stimulating TLR4 and other TLR receptors resulting in the production of inflammatory cytokines [94]. It remains to be proven whether this phenomenon also occurs in humans with depression, which could help explain the observed pro-inflammatory profile. However, systematic reviews in humans suggest that the overall composition of bacterial communities is altered, with certain bacterial taxa being commonly associated with MDD [95–98]. 

It is crucial to understand whether it is possible that commensal bacteria can have an inverse action by alleviating depressive symptoms to open new therapeutic avenues in the treatment of this psychiatric disease. Evidence suggests that peripheral immune activation may lead to changes in central neurotransmitters. Lyte et al. [99] demonstrated that oral administration of the pathogen *Campylobacter jejuni*, at subclinical doses too low to elicit overt immune activation induced anxious behavior in mice. They also reported that the brain stem activation areas, participate in the processing of neural information, leading to autonomic, neuroendocrine, and behavioral responses. It is not clear whether peripherally produced inflammatory cytokines can directly affect the brain, but they were shown to increase the permeability of the blood–brain barrier [100]. Kopp et al. [101] showed that administering *Lacticaseibacillus rhamnosus* GG demonstrated over-regular IL-10 in the plasma of patients. Although IL-10 has potent anti-inflammatory properties, it is thought to act directly as an antinociceptive agent, indicating broad neuroimmune effects, although no impact on behavior has been reported to date. Intestinal microbial balance may thus alter the regulation of inflammatory responses and be involved in the modulation of mood and behavior [102, 103]. Several human studies investigated these issues and found differences in the fecal microbiota of patients with MDD compared to healthy controls [104, 105]. A reduction of *Bifidobacterium* spp. and *Lactobacillus* spp. was observed in 43 depressed individuals [104]. An increase in fecal bacterial diversity was found in a cohort of 46 depressed patients, contrarily to patients who had responded to treatment [105]. In fact, there was an increase in Bacteroidetes, Proteobacteria*,* and Actinobacteria and a decrease in Firmicutes*,* all negatively correlated with the severity of depressive symptoms [105]: while interindividual variability was evident, significant differences were found at the level of genus compared to controls. The administration of a probiotic strain of *Lacticaseibacillus casei* described improvements in mood scores in healthy elderly after treatment, with the greatest benefit for those with a lower baseline mood [106]. Using a multi-strain probiotic (*Lactobacillus acidophilus, Lcb. casei* and *Bifidobacterium bifidum*) on a cohort of MDD patients, improvements in depression scores were observed, as well as beneficial metabolic effects [107]. Another recent open-label study in patients with treatment-resistant depression showed promising results for the probiotic *Clostridium butyrricum* as an adjunct to antidepressant therapy; cognitive performance was further improved by treating patients with MDD with the probiotic *Lactiplantibacillus plantarum* 299v [108]. Overall, systematic reviews of probiotics used as adjunctive therapy in MDD are encouraging and indicate that probiotics are effective in improving mood in humans [109–112], although further clinical studies are needed to strengthen the observed correlations.

### Potential Role of Probiotic, Prebiotic, and Psychobiotic in Reducing the Risk of Depression

As probiotic and prebiotic are capable of modulating and restoring the gut microbiota, their potential role in reducing the risk of depression has been reported [110–112]. Probiotics are living microorganisms that, when administered in adequate amounts, confer a health benefit to the host [113]. In 2017 Markowiak [114] stated that intestinal bacteria are not only commensal but are also subjected to a symbiotic co-evolution together with their host, for which they play a role in modulating the composition of the microbiota, with effects on the epithelial barrier, competing for receptor sites by modulating the expression of tight junctions, producing bacteriocins that inhibit the growth of pathogens, and through the production of SCFA, exerting a trophic anti-inflammatory and protective action of the mucosal barrier. Probiotics colonizing human gut mainly belong to *Lactobacillus*, *Bifidobacterium*, *Lactococcus*, *Sreptococcus*, *Enterococcus*, *Bacillus* and some yeast strains belonging to the genus *Saccaromyces* [114]. Prebiotics are substrates selectively utilized by host microorganisms conferring a health benefit [115]. They belong mainly to three different macro-groups, namely resistant starch, non-starch polysaccharides, resistant oligosaccharides including galatto-oligosaccharides (GOS), fructoligosaccharides (FOS) and xylo-oligosaccharides, which are indigestible to humans but essential for the nourishment of bacteria colonizing our intestines. A further step into the link between probiotics and mental health could be made after the introduction of the term psychobiotic. It was first used by Dinan et al., in 2013 [116] indicating a new class of probiotics with great application potential in the treatment of psychiatric disorders. The species that seemed to be most effective are: i) *Lpb. plantarum (PS128)*, which reduced anxiety and depression in mice [117, 118]; *Lactobacillus helveticus (NS8)* which reduced cognitive dysfunctions linked to anxiety and depression [119]; *Lcb. rhamnosus (JB-1)* which affected anxiety and depression [119].

## Dietary Patterns and Gut Microbiota

**Fig. 2:**
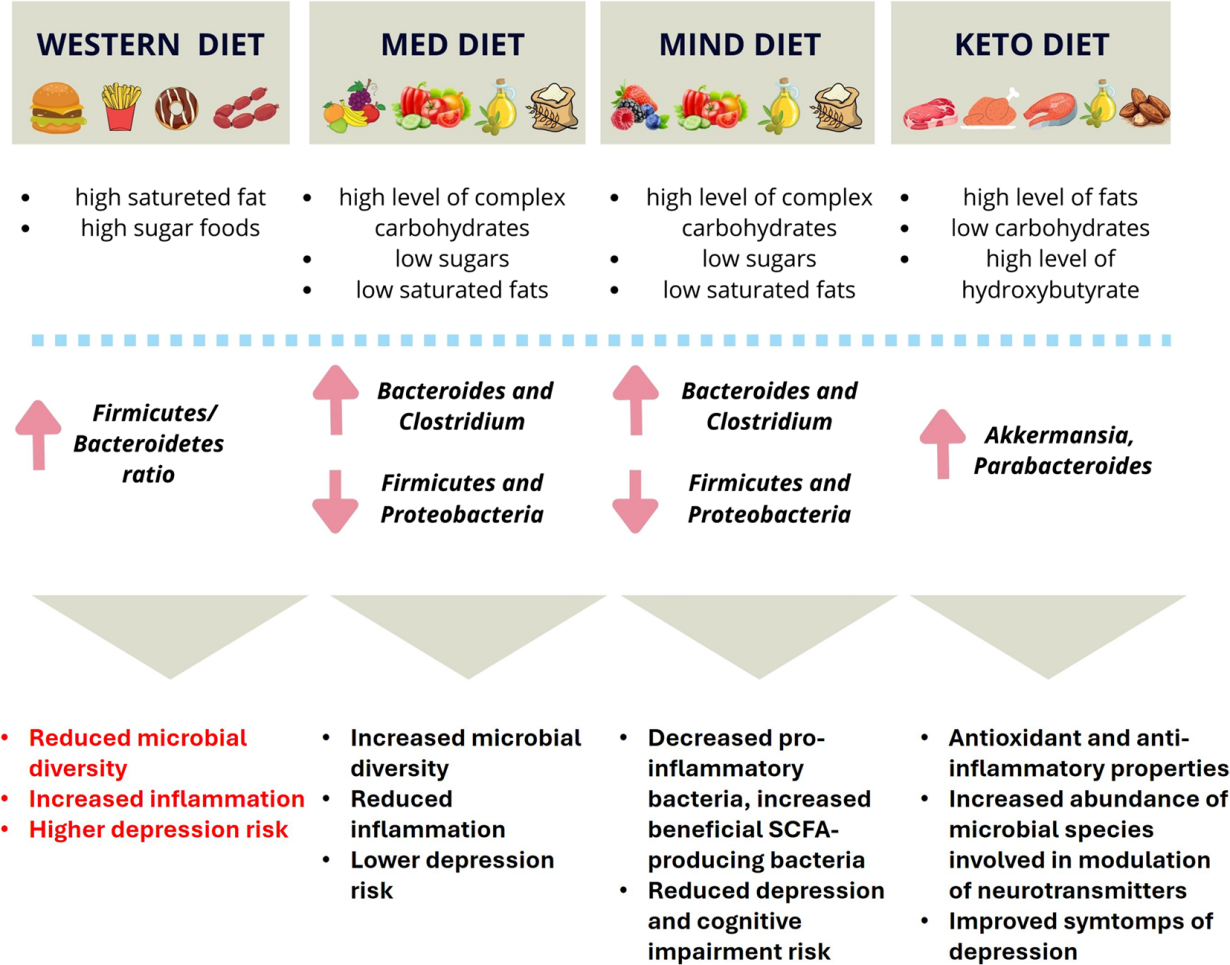
Impact of different dietary patterns on gut microbial composition and main mechanisms possible inferring on mental health. Figure created with Canva

The gut microbial community is likely influenced by diet, which has been reported to play a crucial role in its effects on behavior [120]. The interrelationship between four main dietary patterns and the gut microbiota, possibly impacting on mental health are briefly presented below (Fig. 2).

### Western Diet

Individuals following a Western diet show a gut microbial profile like the one observed in obese individuals [121]. The intake of high fat (HF), high sugars foods (HS), which characterize these types of diets, produces changes in the gut microbial community, a reduced overall microbiota count, a shift in bacteria species abundance, and an overall increase in gut inflammation and permeability [122]. The HFD-driven microbiota composition changes in animal and human models primarily include an increase in the Firmicutes/Bacteroidetes ratio, but specific changes are also due to the considered type of fats and amount of fiber included in the diet [123]. In an animal model study, a HFHS diet caused a significant change in the composition of the intestinal microbiota showing a reduction in Bacteroidetes levels and an increase in Proteobacteria and Firmicutes levels [124]. This was also reported in a similar study in which animals were fed with a high saturated animal fats diet: they showed a significant increase in the abundance of Proteobacteria (*Bilophila wadsworthia*) [125]. *B. wadsworthia* is a member of the human intestine able to use the amino acid taurine in the production of hydrogen sulfide with a demonstrated role in inducing systemic inflammation [126]. Clearly, all these changes at the microbiota level could be able to influence some brain functions and human behavior through patterns described before.

### Mediterranean Diet

The Mediterranean diet consists mainly of cereals (whole grains), nuts, legumes, vegetables and fruits, moderate consumption of poultry and fish [127], and results in some identifiable distinctive traits of the intestinal microbiota [128]. Intervention studies in humans have shown that adherence to a Mediterranean diet can drastically reduce the incidence of neurodegenerative diseases [129–131], psychiatric conditions, cancer [132], cardiovascular disease (CVD) [133–135] and risk of depression [133, 136–138]. The positive impacts of a Mediterranean diet are mediated by its anti-inflammatory potential but are also associated with marked changes in intestinal microbiota, resulting in increased abundance of Bacteroides and Clostridia, a reduction in Proteobacteria and Firmicutes [139] and the related metabolome [140]. A randomized controlled study of dietary intervention to major depressive disorder (SMILES) showed that intervention on the Mediterranean diet (ModiMedDiet) improved scores related to depression [141], demonstrating how the modulation of the microbiota induced by this type of food can also impact on psychiatric diseases. However, further studies are needed to better correlate a Mediterranean diet with its effect on the microbiota-gut-brain axis.

### MIND Diet

The MIND (Mediterranean-Dietary Approaches to Stop Hypertension (DASH) Intervention for Neurodegenerative Delay) diet is a hybrid of the Mediterranean and DASH diets, developed to act specifically on cognitive health in old age [142]. Both the Mediterranean diet and the MIND diet reported positive cognitive outcomes, including prevention of cognitive decline or deterioration and improvement of cognitive performance [143–145]. The MIND diet focuses on increasing the intake of fresh fruits and vegetables and emphasizes the importance of eating foods that are functional for brain function and able to modulate the microbiota-gut-brain axis, such as green leafy vegetables, walnuts, berries, beans, whole grains, fish, poultry, olive oil and wine [142, 146]. On the other hand, foods such as red meats, butter/margarine, cheese, pastries, cakes, and fried or fast foods should be limited [142]. Two high-quality cohort studies reported associations between adherence to the MIND diet and a 53% lower risk of developing Alzheimer’s disease [146] and a slower decline in cognitive function both in general and within specific cognitive domains such as episodic memory, semantics, cognitive velocity and perceptual organization [142]. Interestingly, even modest adherence to the MIND diet was associated with a 35% risk reduction for Alzheimer's disease compared to the lower adherence group. Despite the lack of specific studies on the mechanisms how this diet may impact on the microbiota, outcomes very similar to those derived from the Mediterranean diet are to be expected, considered the common traits of these dietary patterns.

### Ketogenic Diet

The ketogenic diet is a high-fat, low-carbohydrate diet that mimics the metabolic effects of hunger by forcing the body to use primary fat reserves; administration of the ketogenic diet results in increased levels of the ketone bodies hydroxybutyrate, acetoacetate and acetone in peripheral blood and urine [147]. It was designed based on observations on fasting which have been shown to have anti-epileptic properties: the increase of number of ketones in serum has been shown to inhibit apoptotic proteins, improving mitochondrial activity and thus reducing apoptosis in neurodegenerative diseases [148]. This diet mediates the neuroprotective function through the attenuation of oxidative stress and induction of antioxidant protein expression (149), as well as the modulation of the levels of neurotransmitters such as GABA, monoamine and glutamate [150, 151]. Therefore, a ketogenic diet can provide beneficial health effects, improving the symptoms of some neurological conditions, including autism, depression, epilepsy, cancer, as well as Alzheimer's and Parkinson's disease [152–154]. However, the role of the microbiome has recently emerged, considering that the ketogenic diet increases the abundance of *Akkermansia* spp, *Parabacteroides* spp, *Sutterella* spp, and levels of *Erysipelotrichaceae* spp. in the intestinal microbiota in mice compared to the control group [154]. Moreover, colonizing germ-free (GF) mice with strains linked to the ketogenic diet, such as *Akkermansia* spp. and *Parabacteroides* spp., has shown a protective effect against seizures. This is achieved by altering the metabolomic profiles of the colonic lumen, serum, and hippocampus, which are associated with seizure protection [154]. Although ketogenic diet could be considered useful in some specific psychiatric conditions, it cannot be proposed as a large-scale extensible diet due to the low intake of some macronutrients (carbohydrates) and micronutrients that could induce imbalances and metabolic damage in the long run.

## Mechanisms Linking the Gut Microbiota, Diet, and Depression

The mechanisms by which the intestinal microbiome could impact in the pathophysiology of depression [155] through diet, are mainly related to tryptophan metabolism [156, 157], HPA axis [158] and brain-derived neutrophic factor (BDNF) [159]. As stated before, the dominant physiological catabolic pathway for tryptophan is the kynurenine way, where vital neurobiological mediators in a range of neurological and psychiatric disorders [160], including depression [161] and schizophrenia [162] are produced. The onset of this metabolic cascade may be triggered by stress   [163] or by the activation of the immune system and inflammatory pathways [164], making tryptophan availability a crucial factor in mental health management. A variety of foods, including chicken, tuna, oats, peanuts, bananas, milk, cheese, and chocolate contain tryptophan [165]. It is generally absorbed in the small intestine, but significant amounts can also reach the colon, where the intestinal microbiota plays a key role in determining its fate and activity [166, 167]. Direct supplementation of tryptophan has been tested in depressed individuals to improve serotonergic signaling: a review of this studies [168] provided contrasting results. It has been observed that when an activated metabolism of tryptophan is present along the kynurenine pathway, there is an increase in the production of neurotoxic quinolinic acid, leading to opposite effect. The role in the modulation of kynurenine metabolism by dietary interventions other than tryptophan metabolism was also investigated. In vitro and animal models reported individual dietary components such as curcumin [169] and green tea [170], as well as dietary regimens including ketogenic diet [170] and fasting [171], to modulate the activity of the kynurenine pathway. Preliminary intervention studies also suggest that dietary regimens such as calories restriction [172] and individual dietary components such as probiotics, resveratrol, and black tea may also modulate kynurenine metabolism [173, 174].

Diet appears to have an impact also on the HPA axis and clinical intervention studies in healthy adults administered with vitamin C and omega-3 reported reduced cortisol reactivity in response to acute stress [175–177]. Similarly, intervention studies using foods rich in polyphenols, such as pomegranate juice and dark chocolate, reported a reduction in cortisol levels in healthy subjects [178, 179]. Although the mechanisms by which these dietary factors influence cortisol and other measures related to the HPA axis are unclear, this influence may be mediated by modulation of pro-inflammatory response and hypothalamic activation following psychological stress [180]. The hippocampus is a critical component of the limbic system and plays a central role in learning, memory formation, and mood [181, 182]. In mice, increased neurogenesis in the hippocampus is associated with improved learning and memory abilities, while decreased neurogenesis is often associated with depressive behaviors [183]. As already stated, reduced levels of serum BDNF were described in patients with major depression, so much so that protective functions are attributed to BDNF in relation to the pathogenesis of depressive disorders [184, 185]. There is evidence that diet can modulate BDNF and the regulation of hippocampal neurogenesis in adults [186]. Animal models demonstrated that Western-style diets high in fat and sucrose can impair neurogenesis and lower levels of BDNF within the hippocampus negatively impacting cognitive performance [187]. Conversely, research in animal models suggests a beneficial effect of some dietary components, including omega-3 fatty acids, probiotics, and vitamins [188, 189]. It was also shown that single polyphenolic compounds would be able to reverse any adverse events while preserving the integrity of adult hippocampal neurogenesis under conditions of psychopathology, aging, and disease [159]. In the PREDIMED (Prevención con Dieta Mediterránea) study, in a subgroup analysis of participants with depression, participants randomized to a nut-supplemented Mediterranean diet had a higher level of plasma BDNF after 3 years than the control group [190]. However, establishing the relationship between systemic and central BDNF levels is not straightforward, and circulating levels can be affected by different sample processing methods and storage conditions, as well as other peripheral sources of BDNF (e.g., blood platelets) [191]; further clinical studies would therefore be needed to confirm these observations by eliminating any confounding factors.

## Limitations

Despite the comprehensive synthesis of microbiological, nutritional, and neuroscientific evidence, this review has several limitations. First, much of the existing research is based on preclinical or animal studies, which may not fully translate to human populations. The heterogeneity of human microbiota profiles, influenced by genetics, environment, and lifestyle factors, complicates the generalization of findings. Additionally, while the potential of dietary patterns and probiotics in managing depression is highlighted, many studies lack standardized methodologies, such as consistent dosing, duration of interventions, and robust control groups. The complex bidirectional nature of the gut-brain axis also makes it challenging to establish causality in observed associations. Lastly, more longitudinal and large-scale human clinical trials are needed to confirm the therapeutic relevance of microbiota-targeted interventions for depression and to refine dietary recommendations.

## Conclusion

The purpose of this work was to enhance our understanding of the role of microbiota and nutrition in the communication between the intestine and the brain. The vast array of microbes residing in and on the human body appears to influence mental health and disease by affecting this communication pathway. Clinical studies suggest that the diversity and richness of microbiota contribute to resilience, helping to maintain a balanced microbial composition that may facilitate effective interactions between the gut and the brain. Additionally, the potential benefits of probiotics, prebiotics, and psychobiotics in influencing brain function are indicated by both preclinical and clinical research. Nutrition may play a role in supporting microbial balance and influencing the gut-brain axis as it relates to mood and cognition. However, establishing direct correlations between these observations remains challenging due to the complex nature of psychiatric disorders and the variability of individual microbial profiles. Future extensive clinical trials in humans could provide valuable insights into the potential for microbiota-based approaches in the treatment and prevention of psychiatric disorders, possibly offering alternatives to traditional pharmacological methods.

## Data Availability

No datasets were generated or analysed during the current study.
